# Metformin as an Adjuvant Drug against Pediatric Sarcomas: Hypoxia Limits Therapeutic Effects of the Drug

**DOI:** 10.1371/journal.pone.0083832

**Published:** 2013-12-31

**Authors:** Cecilia Garofalo, Mariantonietta Capristo, Maria Cristina Manara, Caterina Mancarella, Lorena Landuzzi, Antonino Belfiore, Pier-Luigi Lollini, Piero Picci, Katia Scotlandi

**Affiliations:** 1 CRS Development of Biomolecular Therapies, Experimental Oncology Laboratory, Rizzoli Orthopedic Institute, Bologna, Italy; 2 Experimental Oncology Laboratory, Rizzoli Orthopedic Institute, Bologna, Italy; 3 Department of Health, University of Catanzaro, Catanzaro, Italy; 4 Department of Experimental, Diagnostic and Specialty Medicine, University of Bologna, Bologna, Italy; University of Navarra, Spain

## Abstract

Metformin, a well-known insulin-sensitizer commonly used for type 2 diabetes therapy, has recently emerged as potentially very attractive drug also in oncology. It is cheap, it is relatively safe and many reports have indicated effects in cancer prevention and therapy. These desirable features are particularly interesting for pediatric sarcomas, a group of rare tumors that have been shown to be dependent on IGF and insulin system for pathogenesis and progression. Metformin exerts anti-mitogenic activity in several cancer histotypes through several molecular mechanisms. In this paper, we analyzed its effects against osteosarcoma, Ewing sarcoma and rhabdomyosarcoma, the three most common pediatric sarcomas. Despite in vitro metformin gave remarkable antiproliferative and chemosensitizing effects both in sensitive and chemoresistant cells, its efficacy was not confirmed against Ewing sarcoma xenografts neither as single agent nor in combination with vincristine. This discrepancy between in vitro and in vivo effects may be due to hypoxia, a common feature of solid tumors. We provide evidences that in hypoxia conditions metformin was not able to activate AMPK and inhibit mTOR signaling, which likely prevents the inhibitory effects of metformin on tumor growth. Thus, although metformin may be considered a useful complement of conventional chemotherapy in normoxia, its therapeutic value in highly hypoxic tumors may be more limited. The impact of hypoxia should be considered when novel therapies are planned for pediatric sarcomas.

## Introduction

The IGF system has an important role in tumorigenesis and cancer progression [1]. In addition, metabolic factors such as obesity and hyperinsulinaemia have been associated with increased overall cancer risk [2]. Although many factors have been postulated to mediate effects of obesity on cancer, recent research has focused on insulin as a potentially relevant mediator [3]. The recognition that the expression of insulin receptors (IR) is not confined to classic insulin-target tissues such as the liver, muscle and fat, but that it extends to normal and transformed tissues raises several ques­tions. The IR is expressed at two isoforms that differ at the carboxyl terminus of the A subunits by 12 amino acids [4]. The IR-B is the classic IR that regulates glucose uptake and binds insulin with high affinity but binds IGFs poorly. Conversely, the IR-A binds both insulin and IGF-2 with high affinity but IGF-1 with low affinity. In some conditions like fetal growth, cancer and diabetes, IR may display some non-metabolic effects like cell proliferation and migration and may affect metastasis and tumor progression. Over-expression of IR-A is in fact emerging as a feature of cancer cells where it mediates cell survival, proliferation, and migration under insulin and IGF-2 stimulus [1], [4], [5]. An autocrine loop involving IGF-2 and IR-A is active in different sarcomas, such as rhabdomyosarcoma and osteosarcoma cells [6], [7], [8]. Recently, we have demonstrated exclusive presence of IR-A in Ewing sarcoma [9]. Moreover, the ratio of IGF-1R:IR-A in favor of IR-A seems to be responsible of native and acquired resistance of some Ewing sarcoma to both monoclonal antibodies and small tyrosine kinase inhibitors (TKI) anti-IGF-1R and it may also explain the lower levels of sensitivity of other sarcomas, such as rhabdomyosarcoma and osteosarcoma to these targeted therapies. In cells resistant to anti-IGF-1R drugs, we observed increased expression of IGF-2 together with increased levels of IR-A; consequently, we presumed these cells undergo a switch from IGF-1/IGF-1R to IGF-2/IR-A dependency to maintain proliferation, migration and metastasis. The proliferative role of IR-A in resistant cells was supported also by increased sensitivity to proliferative effects of insulin while silencing of IR induced inhibition of cell growth [9].

In this perspective, the anti-diabetic drug metformin, a biguanide derivative widely used as first-line pharmacotherapy in non-insulin-dependent diabetes mellitus (T2DM), has recently gained attention in cancer research [10], [11], [12], [13]. The primary systemic effect of metformin is to lower glucose levels through reduced hepatic gluconeogenesis and increase glucose uptake in peripheral tissues such as muscle and fat. Thus indirect benefits of metformin is a decrease in insulin, a growth promoting hormone, suggesting that metformin could affect tumor growth and reduce the risk of cancer. Indeed, epidemiological investigations report that metformin treatment is associated with a decreased incidence of cancers in several organs, such as breast, prostate, colon, and pancreatic cancer [14], [15], [16], [17]. In addition, in clinical settings, metformin improves outcome of diabetic cancers patients, either as single agent as well as in combination with chemotherapeutic drugs, suggesting a potential role on cancer therapy [18], [19]. Metformin was also reported to exert direct effects against cancer cells. At the cellular level, there is considerable evidence showing that metformin partially inhibits complex-I of the respiratory chain in mitochondria, leading to reduced oxida­tive phosphorylation and reduced ATP production. This leads to cellular ATP deficit and activation of AMP kinase (AMPK) which is a cellular energy sensor that downregulates cellular processes that consume energy [20]. In fact, once activated, AMPK restores cellular energy levels by stimulating the catabolic pathway, such as glucose uptake, glycolysis and fatty acid oxidation and stop ATP-consuming processes such as fatty acid, cholesterol and protein synthesis. Furthermore, AMPK activation leads to regulation of multiple downstream pathways involved in the control of cellular proliferation, including inhibition of the mTOR downstream pathway [21]. Although there are still gaps in the understanding of metformin mechanisms of action, mounting evidences indicate an effective role of metformin in cancer therapy.

In the present study we evaluated the *in vitro* and *in vivo* therapeutic effects of metformin against osteosarcoma, rhabdomyosarcoma and Ewing sarcoma, the three most common pediatric sarcomas. In addition, drug-drug interactions between conventional chemotherapeutic agents and metformin were investigated together with the analysis of its anti-proliferative effects in hypoxia conditions.

## Materials and Methods

### Ethics Statement

All animal experiments were authorized by the Experimental protocols approved by the Institutional Animal Care and Use Committee of the University of Bologna, and forwarded to the Italian Ministry of Health (letter 4783-X/10). Mice were treated according to institutional and European Union guidelines.

### Drugs

Metformin, doxorubicin and vincristine was purchased from Sigma, (St. Louis, MO, USA). Actinomycin-D was purchased by Ovation Healthcare International (Dublin, Ireland). The PI3K/mTOR dual inhibitor NVP-BEZ235 was kindly provided by Novartis (Basel, Switzerland)... D-18851, an ifosfamide analog not requiring metabolic activation, was kindly provided by Baxter Oncology GmbH (Frankfurt, Germany). ). Insulin was purchased from Novo Nordisk (Bagsvaerd, Denmark) and IGF-1 from Millipore (Billerica, MA, USA). Working dilutions of all drugs were prepared immediately before use.

### Cell lines

A panel of four osteosarcoma, seven Ewing sarcoma, and three rhabdomyosarcoma human cell lines were analyzed. Saos-2, U-2OS, MG-63, SK-ES-1, SK-N-MC, and RD-ES were provided by American Type Culture Collection, ATCC; the alveolar rhabdomyosarcoma cell line SJ-RH30 was kindly provided by Dr. A. Rosolen (University of Padua, Padua, Italy)[22]; Ewing sarcoma cell lines TC-71 and 6647 were kindly provided by T.J. Triche (Children's Hospital, Los Angeles, CA); the ASP14 cell line, a stable cell line generated from A673 ES cell line transfected with doxycycline(DOX)-inducible shRNA targeting EWS-FLI1 was kindly provided by H. Kovar (St. Anna Kinderkrebsforshung, Vienna). The osteosarcoma IOR/OS10 and the Ewing cell line LAP-35 were obtained in the Experimental Oncology Lab, Rizzoli Institute (Bologna) and were previously described [8], [23]. The RD/18, a clone of the commercially available cell line RD (Flow Laboratories), and the CC-A cell lines were kindly gifted by Prof. P.L. Lollini, University of Bologna and previously characterized [24], [25]. The Ewing sarcoma TC/DOXO8 and the osteosarcoma U-2/DOXO35 were generated by transfection with an expression vector containing full-length MDR1 cDNA and selected in doxorubicin [26], [27]. Cells resistant to anti-IGF-1R agents were obtained from TC-71 Ewing sarcoma cell line, as recently described and characterized [5], [9], and referred as TC/AVE (resistant to AVE1642 MAb, Immunogen Waltham, AM TC/CP (resistant to CP-751,871/Figitumumab, Pfizer, San Diego, CA ), or TC/AEW (resistant to NVP-AEW541, Novartis) All the cell lines have been recently authenticated by STR analysis using genRESVR MPX-2 and genRESVR MPX-3 kits (serac, Bad Homburg, Germany). The following loci were verified: D16S539, D18S51, D19S433, D21S11, D2S1338, D3S1358, D5S818, D8S1179, FGA, SE33, TH01, TPOX VWA. Last control was performed in November 2012. All these cell variants were tested for mycoplasma contamination every 3 months (last control, March 2013) by MycoAlert mycoplasma detection set (Lonza, Nottingham, Ltd). Cultures were grown in a humidified incubator at 37°C with 5% CO2 and maintained in standard medium (Iscove Modified Dulbecco’s medium, IMDM, plus 10% fetal bovine serum, FBS). For hypoxic condition, TC-71 and ASP-14 Ewing sarcoma cells were grown in IMDM containing the hypoxia-mimetic agents cobalt chloride (CoCl2) (200 uM) (Sigma,St Louis, MO, USA).

### Cell culture experiments

To assess cell growth, MTT assay (Roche, Indianapolis, IN) was used according to manufacturer’s instructions. Cells were plated into 96 well-plates (range 2,500–10,000 cells/well) in standard medium. After 24 hours, various concentrations of metformin (1–50 mM) were added and cells exposed up to 72 hours. In combination experiments, cells were treated for 72h with metformin alone (control) or combined in fixed ratio with DXR, VCR, ACT-D and IFO, respectively. For analysis of cell cycle, after 24 to 72 hours of treatment, cell cultures were incubated with 10 µmol/L bromodeoxyuridine (Sigma) for 1 hour in CO2 atmosphere at 37°C and processed accordingly to procedures previously described. For analysis of DNA content and evaluation of apoptosis, cells were fixed with cold 70% ethanol, treated with 0.5 mg/mL RNase, and stained with 20 µg/mL propidium iodide. [28], [29]. Anchorage-independent growth was determined after seeding of 3,300 cells/dish in 0.33% agarose (SeaPlaque, FMC BioProducts, Rockland, ME) with a 0.5% agarose underlay [28].

In combined experiments with IGFs ligands, cells were serum starved for 18h and treated with IGF-I (50 ng/ml) or insulin (10 nM) alone or in combination with metformin (10 mM) for 48h. For protein analysis, starved cells were pre-treated with metformin (10 mM) for 4h and then exposed to IGF-I (50 ng/ml) or insulin (10nM) for 15min.

### Western blotting

Cells were treated with metformin (10 mM) for 30min to 24h or left untreated and cell lysates were prepared and processed as previously described [28]. The following antibodies (Ab) were used: anti-phospho-AMPKα (thr172) Ab, anti-AMPK Ab, anti-phospho S6 (Ser^240/244^) Ab, anti-S6 mAb, anti-ERK pAb, (Cell Signaling Technology, Beverly, MA); anti-phospho-ERK (Tyr202/Tyr204) Ab (Covance, Princeton, NJ); anti-HIF1α Ab, and anti-Beta-actin mAb (Santa Cruz Biotechnology, San Diego, CA, USA). Anti-rabbit or anti-mouse antibodies conjugated to horseradish peroxidase (GE Healthcare, Piscataway, NJ) were used as secondary antibody.

### Real Time PCR

Total IR and IGF-1R was also measured by using the absolute quantification assay, as previously described [9].

### L-Lactate and ATP measurement

Briefly, TC-71, SK-N-MC and 6647 cells were seeded in 6 well or 96 well plates depending on the assay. 24 hours later medium was removed and cells were incubated with either vehicle or metformin (10 mM) for 24–48 h. Following cell treatments, the medium was removed from cells and lactate levels in the extracellular medium were measured using the Lactate Colorimetric Assay Kit (Abcam, Cambridge, MA, USA), accordingly to manufacturer’s instruction. This kit detects lactate levels in samples from concentrations of 0.02–10 mM. Lactate concentration was normalized to sample cell number. Cellular ATP levels were measured using the CellTiter-Glo Luminescent Assay (Promega), accordingly to manufacturer’s instruction.

### Ki-67 staining

Adherent cells grown on coverslips for 48 hours were fixed in cold methanol, blocked with 4% BSA/PBS for 1 hour at room temperature, and incubated with anti-Ki-67 pAb (Santa Cruz Biotechnology, San Diego, CA, USA) (1:50). Polyclonal anti-rabbit FITC (Dako, Glostrup, Denmark) (1:80) was used as secondary antibody. Nuclei were counterstained with Hoechst 33256 (Sigma). Images were taken using Nikon ECLIPSE 90i image analysis system (Nikon, Italy). In each sample, the percentage of Ki-67-positive cells was quantified on at least 500 cells and expressed as Ki-67 labeling index (LI). Images acquisition and processing were conducted using the NIS-Elements A.R. 3,10 software. Acquisition parameters were as follow: NA objective 0.75; R.I. 1, image size: 2560×1920; exp ME:3 sec; noise reduction ON.

### Receptor measurement by enzyme-linked immunosorbent assay

The characteristics and specificity of IR or IGF-1R enzyme-linked immunoabsorbent assays have been previously described [30].

### *In vivo* treatment with Metformin alone or in combination with vincristine

Athymic Crl:CD1-Foxn1 nu (nude) mice, 4–5 weeks old, were purchased from Charles River Italy. For the evaluation of treatment effectiveness, 7 days after TC-71 cells subcutaneous injection, when tumors started to be measurable, mice were randomized into control and treated groups. Mice treated with metformin received the drug either in drinking water (200 mg/Kg, for 3 weeks) or through daily intratumor injection (200 mg/Kg in PBS, 5 days weekly, for 3 weeks) or through daily gavage at 500 mg/kg, 5 days weekly, for 2 weeks. Vincristine was given i.p. (1 mg/Kg/d) on days 7 and 8 after cell injection. For combination therapies, mice received metformin (500 mg/Kg p.o.) and vincristine (1 mg/Kg i.p. on day 7 and 8 after cell injection). The control groups were treated with PBS intratumor or water only p.o. Tumor volume was calculated as π[√(a·b)]^3^/6 where a is the maximal tumor diameter and b is the tumor diameter perpendicular to a.

### Immunohistochemistry

Immunohistochemical staining was done on 3-µm paraffin sections of xenografts derived from *in vivo* experiments. Briefly, dewaxing and antigen retrieval have been obtained using W-CAP TEC BUFFER pH 6 (Bio-Optica, Thermo Fischer Scientific Inc. Fremont, CA) at 98°C for 20–25minutes. Inhibition of endogenous peroxidases was performed in 3% H2O2 solution and the slides were subsequently rinsed in phosphate buffered saline solution (PBS) 1X with 0.01% of detergent Tween-20 (PBS 1X-Tween). All the following incubation steps were performed in humid chamber at room temperature. Sections were incubated for 5 minutes with Ultra V Block solution (Ultravision LP, LabVision Corporation, Thermo Fisher Scientific Inc, Fremont, CA) and then washed in PBS 1X-Tween. They were subsequently incubated for 1 hour with HIF1-alfa (1:700, Novus Biologicals, Littleton, CO), anti-phospho-AMPKα (thr172) (1:20, Cell Signaling Technology) primary antibody and rinsed in PBS 1X-Tween. Sections were further incubated with Primary Antibody Enhancer solution (Ultravision LP, LabVision Corporation) for 20 minutes. After several washes in PBS 1XTween, sections were incubated for 30 minutes with horseradish peroxide–polymer solution (Ultravision LP, LabVision Corporation). Reaction was revealed with Diaminobenzidine (DAB) solution for 3 minutes and counterstained with hematoxylin.

### Statistical analysis

Correlations between two variables were obtained by Spearman’s test. IC_50_ values were calculated from linear transformation of dose-response curves. To define drug-drug interactions (in terms of synergism, additivity, or antagonism), the combination index (CI) of each two-drug treatment was calculated with the isobologram equation [31] by using the CalcuSyn software (Biososoft, Ferguson, MO).

## Results

**Figure 1:**
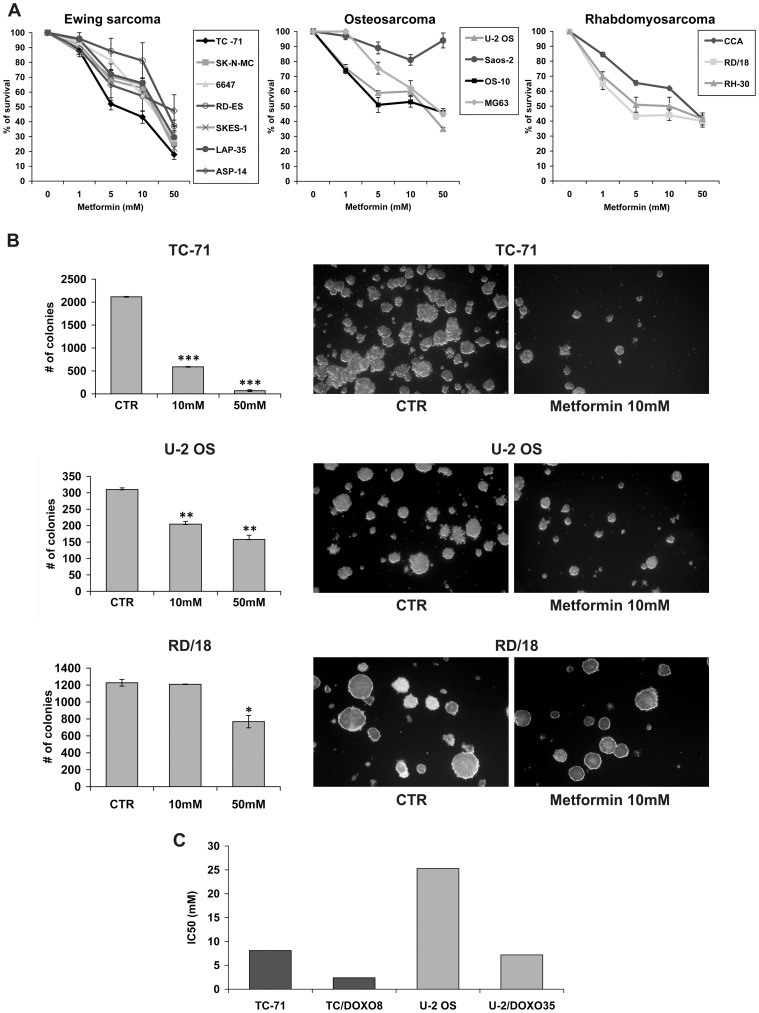
*In vitro* sensitivity of Metformin in sarcomas. **A**) Cell growth was assessed by MTT assay after 72h exposure to Metformin (mM) in seven Ewing sarcoma, four Osteosarcoma, and three Rhabdomyosarcoma cell lines and displayed as a percentage of survival over controls. *Points*, mean of two independent experiments; *bars*, SE. **B**) Effects of metformin on sarcoma cells in anchorage independent conditions. Number of colonies was determined after 7–10 days of growth in IMDM 10% FBS. *Columns*, mean of three independent experiments; *bars*, SE. Bars: mean of three experiments ± SE. *P < 0.05, ** P<0.01, ***P < 0.005 statistically significant differences by Student’s t test. Representative pictures of the effects of the inhibitor on colonies formation in soft agar. Magnification x40; **C)** IC50 values of metformin in Ewing sarcoma (TC/Doxo8) and osteosarcoma (U-2OS/Doxo35) chemoresistant cell lines.

**Figure 2:**
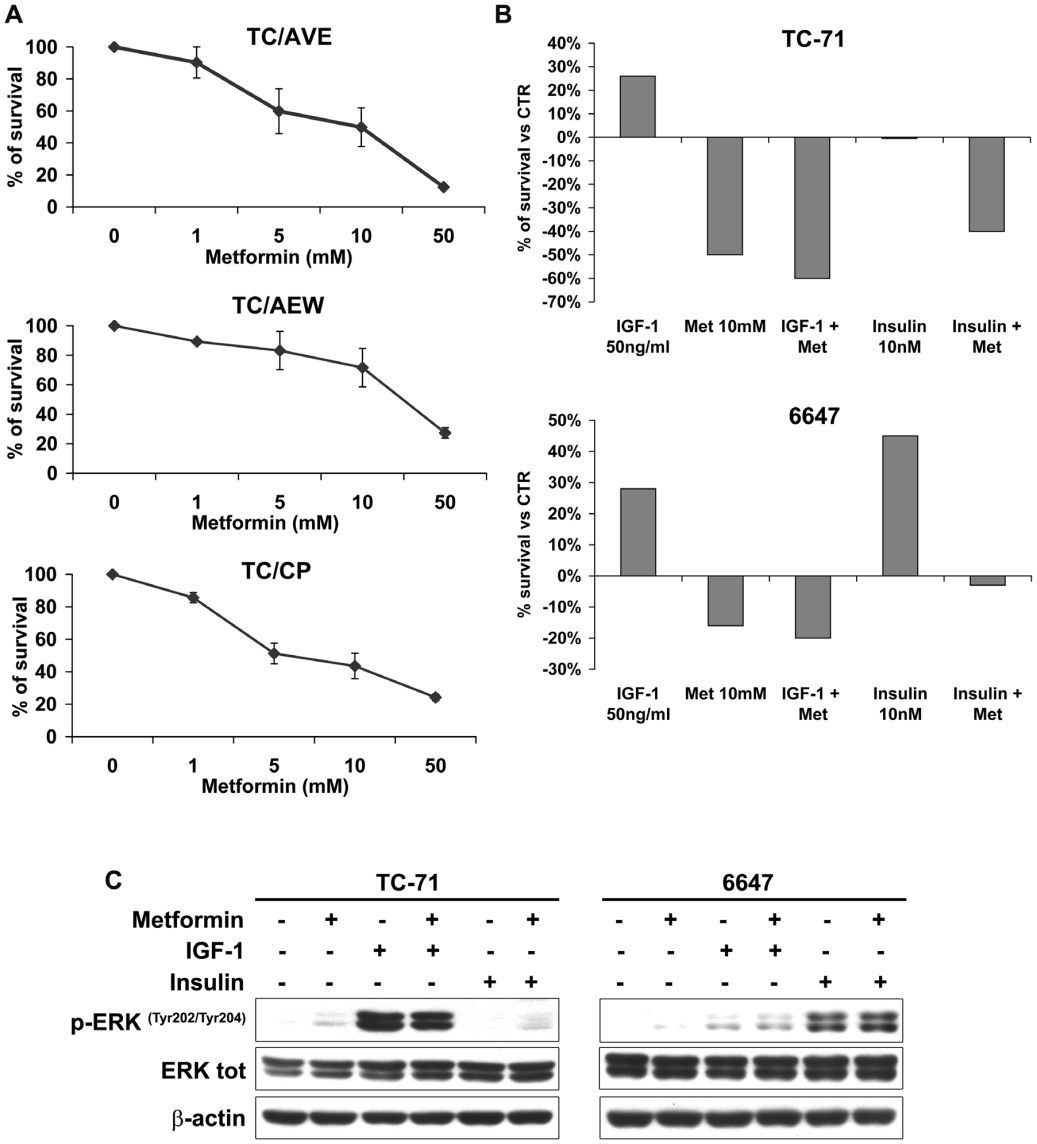
Metformin effects are independent to IGF system activation. **A**) Cell growth was assessed by MTT assay after 72h exposure to Metformin (mM) in cells resistant to anti-IGF-1R agents (TC/AVE, TC/AEW, TC/CP) where recently we demonstrated an increased expression of IR-A with a concomitant downregulation of IGF-1R and displayed as a percentage of survival over controls. *Points*, mean of two independent experiments; *bars*, SE; **B**) After overnight starvation, TC-71 and 6647 EWS cells were exposed to indicate concentration of metformin and/or IGF-1 and/or Insulin in IMDM containing 1% FBS for 48h. Cell growth was assessed by trypan blue assay and shown as percentage of growth over untreated controls. **C**) Starved cells were exposed to metformin (10 mM) for 4h in IMDM plus 1% FBS than stimulated with IGF-1 (50 ng/ml) or Insulin (10 nM) for 15min. After harvesting, cells were lysed and prepared for immunoblot analyses for p-ERK and total ERK. β-Actin was used as loading control.

**Figure 3:**
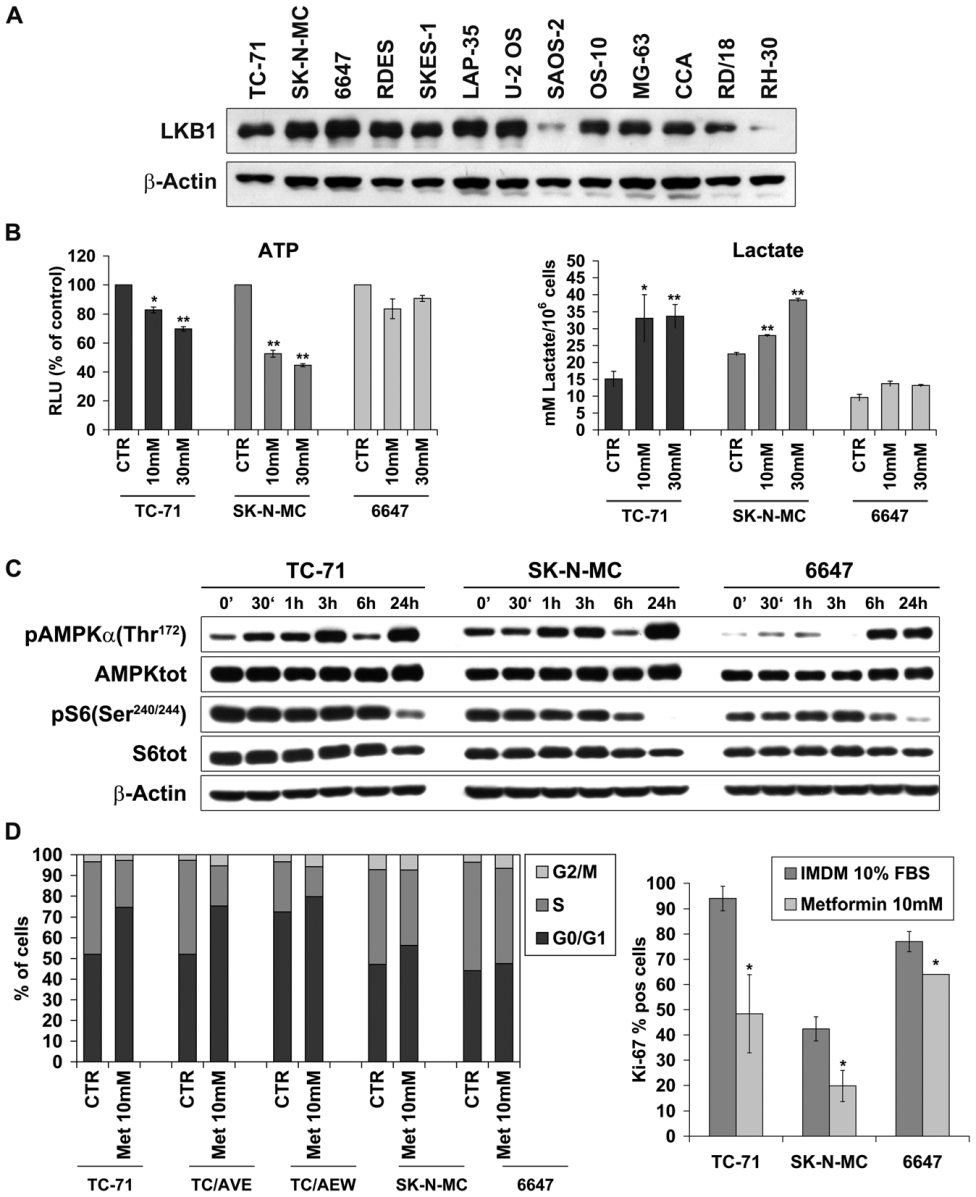
Analysis of metformin targets in Ewing sarcoma cell lines. **A**) protein expression levels of LKB1 in a panel of Ewing and osteosarcoma cell lines. β-Actin was used as loading control. **B**) *Left* Graph showing cellular ATP levels (RLUs) as a percentage of control in control and metformin treated ES cells. *Right* ES cells were treated as described in material and methods, and cellular lactate levels were measured using a colorimetric assay kit (Abcam). Graph shows L-lactate concentration. Values are expressed as mean ± SEM and significance (*) p<0.05; (**) p<0.01 as indicated. **C**) Metformin activity on AMPK and mTOR signaling on Ewing sarcoma cell lines. Western Blotting analysis of metformin-treated cells TC-71, SK-N-MC and 6647. Cells were treated with metformin (10 mM) for the indicated time points (30min-24h). Immunoblot analysis was carried out using antibodies against phosphorylated AMPKα (Thr^172^), AMPKα, phosphorylated pS6K (Ser^240/244^), pS6, and β-actin as normalization. **D**) Analysis of Metformin (10 mM) effects on cell cycle over 48h treatment in three Ewing sarcoma cell lines (TC-71, SK-N-MC, 6647), together with two anti-IGF1R agents resistant cells (TC/AVE, TC/AEW). *Left panel*, mean percentage of cells in different cell cycle phases as determined by flow cytometry analysis; *Righ panel*, Ki-67 positive cells (Ki-67 labeling index). Data are shown as percentages of mean of three independent experiments and significance (*) p<0.05 as indicated.

The effects of metformin on sarcoma cells growth were investigated in a panel of seven Ewing sarcoma, four osteosarcoma, and three rhabdomyosarcoma human cell lines. Ewing sarcoma appeared as the most sensitive histotype when cells were grown in monolayer conditions (Figure 1A). However, also among Ewing sarcoma cell lines metformin was variably effective: TC-71 cells were the most sensitive (IC50 value of 8.1 mM), while RD-ES and 6647 were the most resistant (IC50 value of 39.9 mM and 19.9 mM, respectively). To verify whether metformin is able to affect tumorigenic properties of Ewing sarcoma cells too, we analyzed their anchorage-independent growth by a soft-agar assay using 10–50 nM of metformin. Both doses of metformin gave significant reduction in the number and size of the Ewing sarcoma TC-71 and osteosarcoma U-2 OS colonies, while more limited effects were observed in the rhabdomyosarcoma RD/18 cell line (Figure 1B). The variable efficacy of metformin did not appear to be related to differences in IGFs and insulin pathways. Indeed, no correlation was found between IGF-1R/IR-A expression levels and sensitivity to metformin (Table 1). In addition, in cells resistant to anti-IGF-1R therapies, where we found an increased expression of IR-A [5] together with down-regulation of IGF-1R, the anti-proliferative effects were similar to sensitive cells (Figure 2A). Moreover, when Ewing sarcoma cells were exposed to exogenous IGF1 and/or insulin, metformin prevented IGF-1 and Insulin-induced proliferation and maintained its anti-proliferative effects. TC-71 and 6647 were chosen as representative of a cell line with IGF1/IGF1R or Insulin/IR dependency (Figure 2B). The anti-proliferative effect of metformin was obtained independently from MAPK/ERK pathway, which was induced by ligand exposure and was not affected by metformin (Figure 2C), but rather related to AMPK inhibition (Figure 3). Several reports demonstrate AMPK as a major target of metformin [10], [11], [12], [13]. AMPK may be activated through direct (via increase of AMP/ATP ratio) or indirect (via LKB1-mediated phosphorylation) mechanisms. In our panel of cell lines expression of LKB1 was verified in six Ewing sarcoma, four osteosarcoma, and 3 rhabdomyosarcoma cell lines (Figure 3A) and did not appear to modulate sensitivity to metformin (Table 1). After 24h of metformin treatment, statistically significant dose-dependent decrease in overall intracellular ATP contents was observed in TC-71 and SK-N-MC EWS cell lines, while treatment of 6647 cells, one of the most resistant Ewing sarcoma cells, induced only a modest reduction of the ATP concentration (Figure 3B). When mitochondrial respiration is impaired, cells compensate and increase aerobic glycolysis to improve their bioenergetics. Accordingly, metformin treatment significantly increased L-lactate production in TC-71 and SK-N-MC cell lines confirming the induced glycolytic shift (Figure 3B). These results demonstrate that, in Ewing sarcoma cells, metformin initiates a strong metabolic stress which very likely leads to direct activation of AMPK. Differences in time-dependent activation of AMPK reflect the variable effectiveness of metformin: in TC-71, the most sensitive cell line, metformin induced phosphorylation of AMPKα after 30min of treatment, in SK-N-MC after 1h, while in 6647 we observed increased levels of p-AMPKα after 6 h (Figure 3C). The activated form of AMPK inhibits mTOR activity via the phosphorylation of the tumor suppressor tuberous sclerosis complex 2 (TSC2). As such, western blotting analysis revealed the subsequent inhibition of the S6 protein phosphorylation indicating that conventional targets of metformin are affected in our cells. Based on these data, a combination with therapies directed against PI3K/AKT/mTOR may be an option to potentiate anticancer effects, as recently suggested [32]. Indeed, combination of metformin with BEZ235, a dual mTOR and PI3K inhibitor [33] gave synergistic results in all the three sarcoma histotype (CI<0.9) (Table 2).

**Table 1:** Lack of correlation between IGF-1R and IR expression and sensitivity to metformin in pediatric sarcomas.

HISTOTYPE		IGF-1R a (ng/100 ug of proteins)	IR b (ng/100 ug of proteins)	Ratio IGF-1R:IR	IC_50_ of metformin (mM)	p53 Status
**Ewing Sarcoma**	**TC-71**	2	0.32	6.25	8.1±0.4	p.Arg213X
	**SK-N-MC**	3.69	2.87	1.28	17.4±5.9	c.170_572del
	**6647**	0.81	4.13	0.2	19.9±6.4	p.Ser241Phe
	**RD-ES**	3.74	2.39	1.56	39.9±7.6	p.Arg273Cys
	**SKES-1**	1.82	6.03	0.30	14.5±2.2	p.Cys176Phe
	**LAP-35**	2.38	0.56	4.25	18.8±1.3	wt
**Osteosarcoma**	**U-2 OS**	4.97	0.18	27.61	25.2±2.3	wt
	**Saos-2**	2.38	1.33	1.79	> 50	del2 > EX4-EX8
	**IOR/OS-10**	1.64	5.42	0.30	17.1±1.2	splicing ex9/10
	**MG63**	2.33	0.27	8.6	23.8±2.1	wt
**Rhabdomyosarcoma**	**CCA**	1.56	0.72	2.16	23.8±0.5	nd
	**RD/18**	1.45	1.63	0.90	5.9±2.6	248 Arg-Trp
	**RH-30**	3.20	3.53	0.89	16.3±0.7	273 Arg-Cys

^a^ Spearman’s test r =  0.448, *P*>0.050.

^b^ Spearman’s test r =  –0,402, *P*>0.050.

^c^ Spearman’s test r =  0.457, *P*>0.050.

**Table 2:** *In vitro* combination study of metformin with conventional and targeted drugs in sarcoma cells.

Drug combination	Ewing sarcoma(CI ± SE)			Osteosarcoma(CI ± SE)	Rhabdomyosarcoma(CI ±SE)
	TC-71	SK-N-MC	6647	U-2OS	RD/18
Metformin + Doxorubicin	0.087±0.005	0.344±0.02	0.670±0.007	0.897±0.06	0.997±0.09
Metformin + IFO	0.751±0.05	0.935±0.154	0.902±0.04	0.723±0.06	0.666±0.15
Metformin + NVP-BEZ235	0.589±0.21	0.365±0.08	0.227±0.02	0.564±0.11	0.459±0.122
Metformin + Vincristin	0.507±0.03	0.736±0.02	0.462±0.02	ND	ND
Metformin + Actinomycin-D	0.760±0.03	0.796±0.124	1.738±0.24	ND	ND

Besides inhibiting protein synthesis, AMPK may also alter the process of cell division and cell death. Specifically, metformin was found to decrease the expression of many genes involved in mitosis [34] and may alter cell proliferation or induce cell death depending on the status of p53 [35]. In our cells metformin sensitivity did not vary in relation to p53 status. Most of our cell lines display mutated p53 (Table 1) but no induction of apoptosis was observed after metformin exposure (data not shown). In contrast, in the Ewing sarcoma cells TC-71, SK-N-MC and 6647, showing a truncation (TC-71) or major deletions (SK-N-MC) inactivating p53 transcriptional activity, or carrying the single point mutation S241F in the DNA binding domain which retains part of the wt-p53 activity (6647), metformin treatment led to accumulation of cells in G1 phase (Figure 3D). Effects on cell cycle were similar in cells sensitive or resistant to anti-IGF1R drugs, again sustaining that metformin effects were independent from IGF1R or IR expression. The degree of G1 cell accumulation as well as of cell proliferation inhibition, as shown by Ki-67 staining (Figure 3D), reflects the different sensitivity of TC-71, SK-N-MC, 6647 cells to metformin, further confirming that in the most sensitive sarcoma histotype metformin mainly displays a cytostatic effect. As a consequence, to be therapeutic relevant metformin should be associated with chemotherapy. Combined treatments with vincristine, doxorubicin, actinomycin-D and ifosfamide, the main drugs currently used in the therapy of sarcoma patients [36], [37], showed that metformin produced significant increases in the efficacy of chemotherapeutic agents, with synergistic or additive effects (Table 2). Despite previous evidences showed a role of p53 status in the response to drug-drug combination [38], [39], we failed to observe a such a relationship in our cells. Growth inhibitory effects of metformin were also confirmed in chemoresistant sarcoma cell lines (Figure 1C). Metformin was active either in Ewing sarcoma (TC/DOXO8) or osteosarcoma (U-2/DOXO35) cells resistant to multiple drugs (e.g. doxorubicin, epirubicin, vincristine) [26], [27] confirming its therapeutic potential in the management of sarcomas.

**Figure 4:**
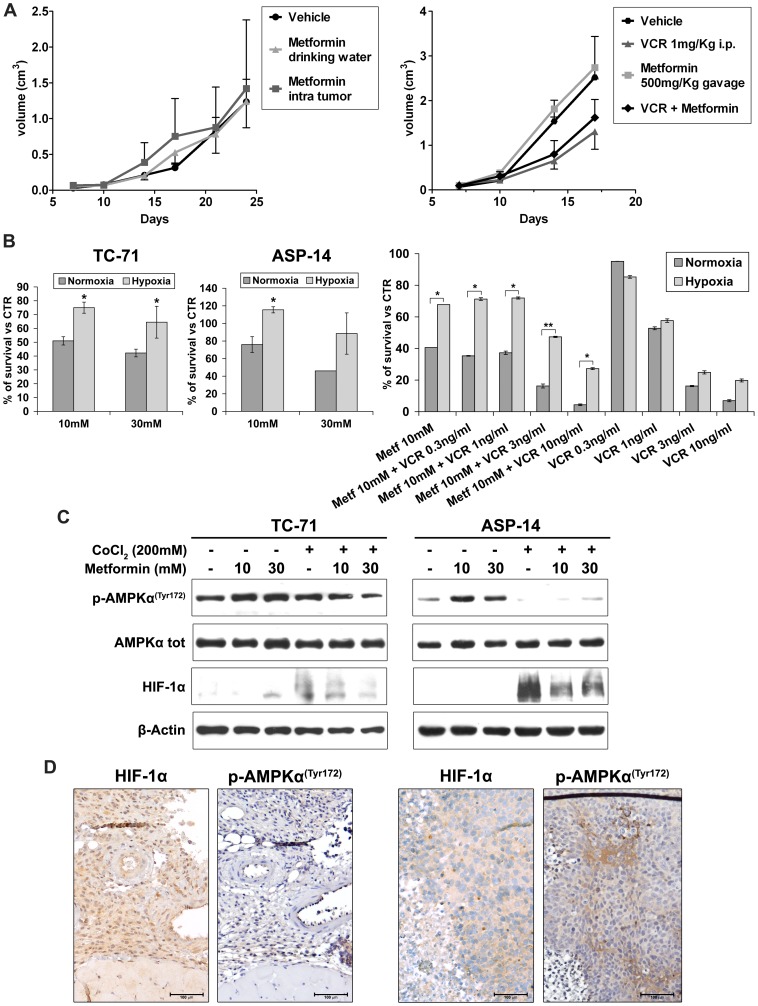
Hypoxia influence the effect of metformin on Ewing sarcoma. **A**) *In vivo* treatment with metformin in association or not with vincristine (VCR) against TC-71 xenografts in athymic nude mice. Treatments began when tumors started being measurable at day +7 after subcutaneous (s.c.) cell injection. *Left* graph, *in vivo* growth curves of TC-71 tumor volume (cm3) after s.c. injection of 5×10^6^ TC-71 cells in groups of 5 mice (treated groups) or 8 mice (Vehicle group). Vehicle, mice treated with PBS intratumor or water alone; metformin in drinking water, mice receiving metformin 200 mg/Kg in drinking water; metformin intratumor, mice receiving intratumor injection of metformin 200 mg/Kg daily, 5 days weekly. *Right* graph, *in vivo* growth curves of TC-71 tumor volume after s.c. injection of 7.5×10^6^ TC-71 cells in groups of 5 mice. Vehicle, mice treated with PBS; Metformin, mice treated daily, 5 days weekly with 500 mg/kg Metformin p.o. (gavage); VCR, mice treated with 1 mg/kg/d i.p. for 2 consecutive days; Metformin+VCR, combined therapy with Metformin 500 mg/Kg p.o. and VCR 1 mg/Kg/d i.p. **B**) Decrease responsiveness to metformin in hypoxia respect to normoxia environment. In hypoxia experiments, TC-71 and ASP-14 Ewing sarcoma cells were pre-treated for 18h with hypoxia mimetic agent CoCl2 (200 uM) than exposed for 48h to different concentration of metformin (10–30 mM) or left untreated. In combination experiments, TC-71 Ewing sarcoma cells were pre-treated for 18h with CoCl2 (200 uM) than exposed for 48h to metformin (10 mM) alone or in combination with different concentrations of vincristine (0.3–10 ng/ml). Effects of metformin on cell growth were assessed by trypan bleu assay and shown as percentage of survival over untreated control. *P < 0.05, ** P<0.01 statistically significant differences by Student’s t test. **C**) Western blotting analysis of AMPKα phosphorylation levels under hypoxia and normoxia conditions after metformin (10–30 mM) treatment. TC-71 and ASP-14 EWS cells were pre-treated for 18h with CoCl2 (200 uM) than exposed for 4h to different concentration of metformin (10–30 mM) or left untreated. Accumulation of HIF-1α protein confirmed the induction of hypoxia; β-actin was used as loading control. **D**) Immunohistochemical evaluation of HIF-1α and phospho-AMPKα in hypoxic (left panel) and normoxic areas (righ panel) in xenografts. Representative figures are shown (magnification X100).

The synergistic *in vitro* effects with vincristine led us to verify the efficacy of metformin also *in vivo* against TC-71 xenografts. Despite the significant effects observed *in vitro*, we failed to observe any reduction of tumor growth when metformin was used alone. Mice bearing tumors 7 days after cell injection were treated with metformin. Either injection near the tumors or oral administration of metformin lacked to give tumor growth reduction (Figure 4A). At the highest dose (500 mg/Kg) oral administration of metformin was unable to inhibit tumor growth, or it even seems to increase it. In addition, combination with vincristine lacked to give any advantage with respect to vincristine alone (Figure 4A).

To understand these discrepancies between *in vitro* and *in vivo* data, we analyzed the possible impact of hypoxia on metformin efficacy in Ewing sarcoma cells. Hypoxia is an important condition in the tumor cell microenvironment. Robust tumour growth requires the presence of a local vascular network that supplies both oxygen and nutrients to tumour cells. However, a highly proliferating mass of tumour cells, such as Ewing sarcoma, develops faster than the vasculature, and tumour cells rapidly meet up with an avascular environment deficient in oxygen, i.e. hypoxic. A large fraction of primary Ewing sarcoma accumulates the hypoxia-inducible factor HIF-1α [40], [41] a key transcription factor that regulates genes that are exploited by tumour cells for survival, resistance to treatment and escape from a nutrient-deprived environment [42], presumably reflecting hypoxia induction in those tissues. In TC-71 xenografts, we could indeed confirm the presence of several hypoxic regions, as shown by nuclear accumulation of HIF-1α (Figure S1). We thus determined whether the culture environment (normoxia or hypoxia) influenced the effect of metformin on Ewing sarcoma viable cell number. TC-71 and ASP14, a cellular variant of A673 EWS cell line reported to have a functionally HIF-1alpha activated pathway in response to hypoxia [43], were grown in normal or hypoxia conditions and exposed to metformin alone or in association with vincristine. Both cell lines were statistically significantly less sensitive to metformin as well as to metformin plus vincristine in hypoxia compared to normoxia (Figure 4B). Stabilization of HIF-1α protein confirmed the hypoxic culture conditions in these experiments (Figure 4C). Hypoxic conditions counteracted the inhibitory effects of metformin possibly by preventing the metformin–induced phosphorylation of AMPK (Figure 4C). This appeared to be confirmed in vivo. We could observe a great heterogeneity with respect to phosphorylation of AMPK inside xenografts treated with 500 mg/kg/die of metformin. Quite interestingly, the activation of the kinase was observed only in the areas that were found to be negative for HIF-1α expression. In contrast, in the hypoxic regions, as indicated by HIF-1α positivity, we did not observe the phosphorylation of AMPK (Figure 4D). This observation is only explorative and need validation but confirms our in vitro evidences, supporting the need of deeper studies comparing activity of metformin in normoxia and hypoxia.

## Discussion

Development of new complementary biomolecular approaches helping to keep treatment-related toxicity to a minimum and to optimize systemic-disease control is still an urgent need for patients with sarcomas. Scientists have tried to fill this therapy vacuum by working on identification of new therapeutic targets, including components of the IGF system [44]. However, development of drugs is difficult for rare tumors and lack of novel drugs strongly has limited any substantial improvements in the field. Metformin may represent a possibility that deserves to be explored, particularly in pediatric sarcomas. The drug is inexpensive, relatively safe and can be potentially used in rational combinations with other drugs, as an adjuvant therapy to potentiate conventional and targeted agents [10], [19], [45]. There are no doubts that IGF system is important for the pathogenesis and progression of sarcomas [46]: cells produce ligands and express the receptors creating a complex network of autocrine stimulations that may sustain tumor growth based either on IGF1/IGF1R or IGF2/IR axis [5], [9]. In this paper, we demonstrated that metformin was equally effective in sarcoma cells sensitive or resistant to different drugs, including conventional or anti-IGF1R agents. In addition, metformin was still able to inhibit sarcoma cell growth in the presence of IGFs, a fact particularly relevant for bone tumors that are exposed to high concentrations of these factors either through autocrine tumor production or IGFs release from bone matrix during tumor growth and bone disruption. Thus, metformin appears as a very attractive adjuvant drug in vitro. Among the three most common solid tumors in pediatric age, Ewing sarcoma was confirmed to be the most sensitive one, in keeping with its higher sensitivity to agents affecting IGF system [47], [48]. However, also among Ewing sarcoma cell lines, the level of sensitivity to the drug varied remarkably. Understanding the link between genetic variations and response to drugs would be essential to move towards personalized treatment. Metformin requires the organic cation transporters such OCT1/2 to be transported into cell, whereas its extrusion is facilitated by the multidrug and toxin extrusion 1 protein (MATE1). Polymorphisms or mutations in the genes encoding for these transporters have been described and may affect metformin effects as well as tumor LKB1 expression [49]. In our cells, we observed that metformin inhibited tumor cell proliferation independently of the levels of LKB1 but rather through an increase of AMP/ATP ratio which leads to direct dose-dependent activation of AMPK and subsequent inhibition of mTORC1, inactivation of S6 kinase and general reduction of protein synthesis. Through these events, and in relation to the level of inhibition in the different cells, metformin reduces cell growth and induces cell-cycle arrest at G1 phase. Despite that most of sarcoma cell lines display deficient p53 functions [50] metformin fails to induce apoptosis, thus suggesting that the drug may have clinical value in combination with other agents rather than alone. Given its inhibitory effects on mTOR signaling pathway, metformin may indeed serve as a chemosensitizer with respect to different type of drugs. Several reports showed that metformin may cooperate with different chemotherapeutic agents to increase their anticancer activity and provided evidence for a convergence of metformin and drugs at the level of AMPK [45]. Through AMPK-mediated inhibition of IRS-1 phosphorylation, metformin participates to the inhibition of the IGF/insulin pathway and shows potential advantages with respect to other mTOR inhibitors, such as rapamycin [51], [52]. This effect may be particularly relevant for pediatric sarcoma. S6K was found to be a crucial modifier of anti-IGF-IR drugs activity [53], [54] and the anti-IGF1R MAb Cixutumumab combined with the mTOR Inhibitor Temsirolimus showed evidence of durable antitumor activity in heavily pretreated EWS family tumors [55]. Here we showed synergistic preclinical effects through combination of the dual inhibitor mTOR/PI3K BEZ235 and metformin, confirming with a cost-effective drug that this molecular targeting association may be effective against patients that were previously found to be resistant to targeted therapies through upregulation of mTOR and/or ERK pathways [56]. In addition, accordingly with data obtained in other tumors [18] metformin was found to potentiate the *in vitro* antimitogenic activity of doxorubicin and vincristine against sarcoma cells. The antiproliferative effect of metformin was even found in doxorubicin-resistant variants of osteosarcoma and Ewing sarcoma cells. This effect together with recent evidence that metformin may protect against doxorubicin-induced cardiotoxicity [57] and that it inhibits P-glycoprotein expression [58], one of the major adverse biomarkers in osteosarcoma [59] further supported its potential use as adjuvant drug. However despite these encouraging results there are still some concerns regarding the use of metformin. One of the main limitations in interpreting the many in vitro studies is related to its concentration[12]. It is certainly true that in vitro metformin is generally used at doses well above the feasible therapeutic plasma levels in humans [60]. However, it is necessary to consider that in vitro cells are conventionally maintained in non-physiological conditions, being exposed to excessive concentrations of insulin, glucose and growth factors which may account for the required elevated doses of metformin. In addition, cells in vitro exposed to metformin do not benefit from the indirect systemic effects of the drug on peripheral tissues, which result in decreased circulating levels of insulin and IGFs [10]. Further studies on more physiological models will be required before establishing whether and how metformin may be used in clinical settings. In vivo studies may be of help being able to give a more comprehensive view of the mechanisms of action of metformin. However, in contrast with what reported in xenografts generated with prostate and lung cancer cell lines [19], neither metformin injection near the tumor nor oral administration were able to decrease Ewing sarcoma tumor growth. Oral administration of metformin was found to be ineffective also in combination with vincristine despite the synergistic effects observed *in vitro*. Reasoning on so different metformin effects in vitro and in vivo we decided to explore the possible involvement of hypoxia, a major feature of solid tumors. Decreased availability of oxygen was indeed reported to increase patient treatment resistance and favor tumor progression [61]. Mainly through activation of HIF1-alpha, hypoxia promotes angiogenesis and initiates a cascade of events that allows tumor cells to continue to proliferate. It was recently shown that Ewing sarcoma cells adapt to hypoxia by redefining their transcriptome and acquiring a distinct hypoxic phenotype characterized by increased invasiveness and anchorage-independent growth [41]. EWS-FLI1, the genetic hallmark of Ewing sarcoma [62], and HIF-1α were found to collaborate in both synergistic and antagonistic ways to regulate cellular metabolism under hypoxia. Interestingly, the majority of EWS-FLI1 regulated genes showed opposite transcriptional effects in normoxia or hypoxia conditions, such as the survival factor, IGFBP-3, which has previously been shown to be suppressed by EWS-FLI1 in normoxia but highly upregulated under hypoxia or by the pro-apoptotic gene BAX, strongly downregulated in hypoxia [41]. As recently reported, these changes may contribute to HIF-1α dependent protection of Ewing cells from anti-cancer drug- or tumor necrosis factor-related apoptosis-inducing ligand (TRAIL)-induced apoptosis [43], [63]. In addition, hypoxia has a major effect on cell metabolism. Through the increase in HIF-mediated expression of both glucose transporters and enzymes of the glycolytic pathway, hypoxia contributes to favor cytoplasmic conversion of pyruvate to lactic acid and glycolysis. In Ewing sarcoma, the tumor-associated chimeric gene EWS-FLI1 is known to induce the accumulation of hypoxia-induced transcription factor HIF-1α [41] and cells have been recently shown to simultaneously activate mitochondrial respiration and high levels of glycolysis [64]. It is thus possible that while in normoxia the metformin- induced glycolytic shift is able to generate a stress sufficient for cells to induce direct activation of AMPK, in hypoxia the accumulation of HIF1 may make respiration more efficient [65] and protect cells from damages, including energy perturbations induced by metformin. Besides confirming previous evidence which showed how hypoxia may be amajor factor in the tumor microenvironment of Ewing sarcoma [41], [43], [63], our findings suggest a note of caution in the general enthusiasm that is linked to clinical use metformin and other biguanides. Although the effects of metformin in tumor prevention seem to be really encouraging, its efficacy in tumor therapy is still under evaluation. Mechanisms of action are still incompletely defined and require deeper cellular-context definition. In this paper we suggest that the impact of hypoxia should be also considered to obtain full exploitation of the remarkable advantages of this clinically attracting drug.

## Supporting Information

Figure S1 **Immunohistochemical evaluation of HIF-1alpha in xenografts**. Representative figures are shown (magnification X100). Enlargement of a detailed section is shown to highlight nuclei staining (magnification X200 and X400).
(TIF)
