# Supplementary figures and images for: Metformin as an Adjuvant Drug against Pediatric Sarcomas: Hypoxia Limits Therapeutic Effects of the Drug
Source: PLoS One. 2013 Dec 31;8(12):e83832. doi: 10.1371/journal.pone.0083832 (PMC3877110; doi:10.1371/journal.pone.0083832)

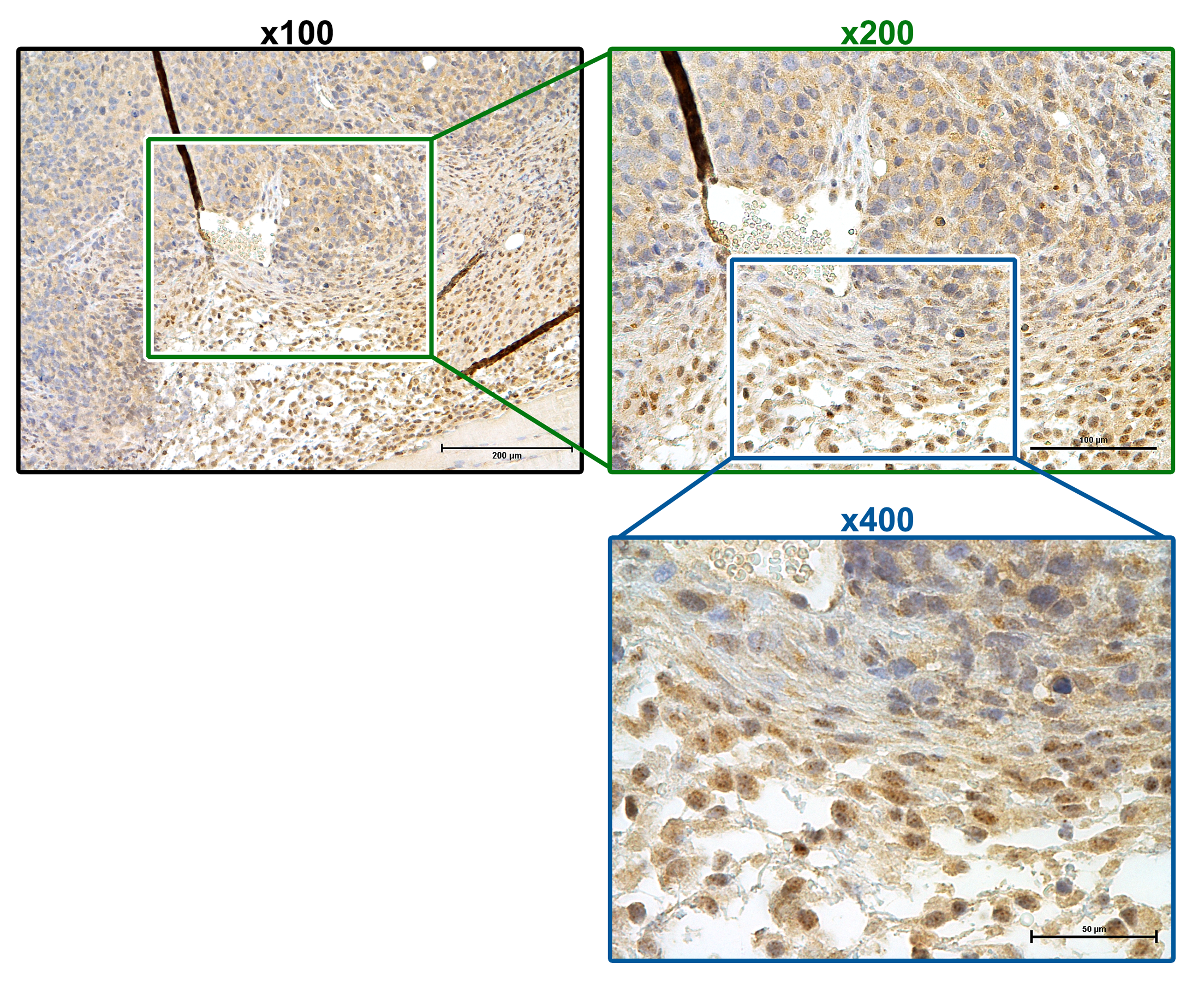

Supplement: Figure S1 — Immunohistochemical evaluation of HIF-1alpha in xenografts. Representative figures are shown (magnification X100). Enlargement of a detailed section is shown to highlight nuclei staining (magnification X200 and X400). (TIF) [file pone.0083832.s001.tif]
